# Safety and Efficacy of Tolvaptan for Acute Refractive Hyponatremia Associated with Traumatic Brain Injury

**DOI:** 10.3390/jcm14176138

**Published:** 2025-08-30

**Authors:** Shashvat Desai, Kathleen Holsaeter, Alexandra Winski, Jeffrey F. Barletta, Frank Bauer

**Affiliations:** 1 HonorHealth, Scottsdale, AZ 85258, USA; shdesai@honorhealth.com (S.D.); kholsaeter@honorhealth.com (K.H.); awinski@honorhealth.com (A.W.); 2 College of Pharmacy, Midwestern University, Glendale Campus, Glendale, AZ 85308, USA

**Keywords:** hyponatremia, tolvaptan, vasopressin antagonists, TBI, SIADH

## Abstract

**Background:** Hyponatremia, defined as a serum sodium concentration below 135 mmol/L, is a common and serious electrolyte disturbance in patients with traumatic brain injury (TBI), and may be treated with vaptans—vasopressin receptor antagonists that promote water excretion. This study evaluates the safety and efficacy of tolvaptan, a vaptan, in correcting hyponatremia in TBI patients compared to a non-trauma cohort. **Methods:** We conducted a single-center retrospective analysis of 126 adult patients in the intensive care unit who received tolvaptan. The study included 73 TBI patients and 53 non-trauma patients with chronic medical conditions. Serum sodium levels were assessed 48 h after tolvaptan administration and compared between the two groups. **Results:** At baseline, the mean sodium level was higher in the TBI group compared to the non-trauma group (128.3 ± 4 mmol/L vs. 125.3 ± 5 mmol/L, *p* = 0.003). Both groups showed a significant increase in sodium levels after 48 h of tolvaptan therapy, and while the post-treatment sodium level was higher in the TBI group, the absolute change was not significantly different between the two groups (132.3 ± 5 mmol/L vs. 130.9 ± 7 mmol/L, *p* = 0.18). Sodium normalization (135–145 mmol/L) occurred in 48% of TBI patients versus 30% of non-trauma patients (*p* = 0.045), though this difference was not statistically significant after adjusting for baseline sodium levels. No cases of osmotic demyelination syndrome were observed. **Conclusions:** Our preliminary analysis suggests that tolvaptan effectively increases sodium levels in both TBI and non-trauma patients with hyponatremia. Further research is needed to fully characterize this response and determine the optimal use of tolvaptan for managing hyponatremia in the TBI population.

## 1. Introduction

Hyponatremia, defined as a serum sodium level of below 135 mmol/L, is the most frequent electrolyte abnormality encountered in hospitalized patients and is associated with increased morbidity and mortality [1]. In patients with traumatic brain injury (TBI), hyponatremia can be exceptionally dangerous due to the potential for cerebral swelling, increased intracranial pressure, and impaired neurological recovery [1]. A recent meta-analysis showed that hyponatremia is associated with increased mortality, prolonged ICU stays, and poorer neurological outcomes in TBI patients [2]. Additional observational data have linked the presence and severity of hyponatremia with increased risk of complications, including cerebral edema and delayed recovery [3,4]. The most common etiology of hyponatremia is the syndrome of inappropriate antidiuresis (SIADH), for which fluid restriction has been the first-line treatment. However, this intervention is often insufficient [5].

Vaptans, non-peptide antagonists of arginine vasopressin (AVP) receptors, selectively increase solute-free water excretion from the kidneys and have been shown to be useful for the treatment of hyponatremia [6]. One study by Jeon et al. (2013) demonstrated the safety and efficacy of oral vasopressin V2 receptor antagonists in patients with acute brain injury in neurocritical care patients with SIADH [7]. Among this class of medications, several subsequent studies have studied tolvaptan, including systematic reviews and a meta-analysis, which have demonstrated that it is both effective and safe in treating hyponatremia due to SIADH with a low risk of overly rapid correction [8,9,10,11]. Additionally, the United States Food and Drug Administration (FDA) has approved tolvaptan for the treatment of euvolemic hyponatremia, including those with SIADH, and hypervolemic hyponatremia, such as those with heart failure and chronic kidney disease after several studies and a case report showed tolvaptan improved outcomes, sodium levels, and was a safe alternative [12,13,14,15]. Data have also been promising for those with hyponatremia in the setting of more of an acute illness, such as malignancy and postsurgical SIADH [16,17,18,19]. Furthermore, other research suggests a potential role of tolvaptan in correcting hyponatremia in the neurocritical care setting [20,21]. However, robust data are still lacking, particularly using tolvaptan in those with traumatic brain injuries [22,23]. However, this medication may be a significant life-saving measure for patients with TBI and SIADH.

Given the scarcity of large-scale studies evaluating both the safety and efficacy of tolvaptan in TBI patients, this investigation seeks to fill that gap. Understanding the effect of tolvaptan and elucidating optimal treatment strategies requires further research. Our institution has been utilizing cases of refractory hyponatremia (persistent hyponatremia despite primary therapy for 24 h) associated with TBI when standard measures have failed. The aim of this study is to perform a single-center, retrospective analysis of the utility of tolvaptan for the correction of hyponatremia due to SIADH in patients with TBI and compare these outcomes with a non-trauma cohort. We hypothesize that tolvaptan is both effective and safe in this critically ill population and may offer a practical therapeutic option for managing refractory hyponatremia in patients with traumatic brain injury.

## 2. Materials and Methods

After local Institutional Review Board approval, we performed a retrospective review of the inpatient database of adult ICU patients at a community-based, level-one trauma hospital in Arizona (HonorHealth Osborn Medical Center, Scottsdale, AZ, USA) between January 2019 and May 2024. Demographic, clinical, and treatment data were collected by systematic chart review using Epic^®^ electronic medical record system. Biochemistry, radiology data, and patient outcomes were collected and analyzed. 

### 2.1. Patient Selection

All patients who received tolvaptan during the defined study period were identified based on querying electronic health records for those who had been administered tolvaptan during the defined study time period, and these patients were screened for inclusion and exclusion criteria. Inclusion criteria consisted of patients aged 18 years or older in the intensive care unit, documented serum sodium level below 135 mmol/L prior to tolvaptan administration, administration of tolvaptan for the treatment of hyponatremia during the study period, and availability of complete medical records for retrospective data collection. Exclusion criteria consisted of patients whose sodium levels improved/normalized with other treatments and those not in the intensive care unit. Two reviewers independently screened patient charts, with discrepancies resolved by a third reviewer. Patients with traumatic brain injury were screened with labs and urinalysis, including serum sodium and urine sodium. Patients with a clinical diagnosis of traumatic brain injury, serum sodium less than 135 mmol/L, and urine sodium greater than 20 mmol/L were included in this study.

### 2.2. Tolvaptan Administration Protocol

In both groups, tolvaptan was started after initial management strategies for hyponatremia proved insufficient. Fluid intake, output, and daily fluid balance were recorded to assess volume status and ensure the absence of hemoconcentration. These data supported the diagnosis of euvolemic hyponatremia consistent with SIADH in all patients prior to tolvaptan initiation. For the TBI group, initial management included free water restriction, urea powder, hypertonic saline, and/or salt tablets. Tolvaptan was considered if serum sodium levels did not improve significantly by 48 h despite conservative measures. All other forms of medical management were ceased before proceeding with tolvaptan use. In the non-trauma group, similar to the TBI group, initial medical treatments for hyponatremia associated with chronic conditions were attempted first. Tolvaptan was initiated if these treatments failed to achieve adequate correction of sodium levels. Tolvaptan was administered as a 15 mg single dose orally. Repeat dosing was at the discretion of the provider. Sodium levels were then documented 24 and 48 h later. All medical treatment was stopped prior to initiating tolvaptan. 

### 2.3. Data Collection

Following approval by the Institutional Review Board (IRB), electronic health records were queried to identify patients receiving tolvaptan during the defined study period. Following that, a systematic chart review was conducted by two independent researchers to collect relevant clinical laboratory data. Demographic variables included age, diagnoses, and injury severity score (for trauma patients). Medical history was reviewed for pre-existing conditions, including chronic kidney disease (CKD), congestive heart failure (CHF), and liver disease. Biochemical data collected included serum sodium levels both before tolvaptan administration (pre-tolvaptan) and 24 and 48 h after tolvaptan administration (post-tolvaptan), as well as serum and urine osmolality. Adverse events were also documented, with particular attention to the occurrence of osmotic demyelination, which was monitored clinically with a provision to perform imaging to confirm diagnosis. This information was then entered into a spreadsheet.

### 2.4. End Points

The primary efficacy outcomes included the rate of successful correction of hyponatremia, defined as achieving a serum sodium level ≥135 mmol/L within 48 h of tolvaptan administration, in both the TBI and non-trauma groups. Additional efficacy measures included the mean change in serum sodium levels from baseline (pre-tolvaptan) to 48 h post-tolvaptan administration in both groups, as well as the proportion of patients who demonstrated any increase in serum sodium levels within 48 h of the initial dose. The primary safety outcome was the incidence of osmotic demyelination syndrome identified through clinical and radiological findings. 

### 2.5. Statistical Analysis

Baseline characteristics, efficacy outcomes, and safety outcomes were analyzed and compared with patients in the trauma and non-trauma groups of patients. Continuous variables are reported as mean ± SD or median (IQR) as appropriate. Categorical variables are reported as proportions. Between-group comparison for continuous variables was conducted using the Student *t*-test and categorical variables using the χ^2^ test or Fisher’s exact test, as appropriate. Significance was defined as a *p*-value of less than 0.05. Multivariate analysis was performed using the covariates of the indication for tolvaptan and baseline sodium levels to account for differences in baseline values between groups. Statistical analysis was undertaken using IBM SPSS Statistics 23 (IBM-Armonk, New York, NY, USA).

## 3. Results

### 3.1. Baseline Characteristics

**Figure 1:**
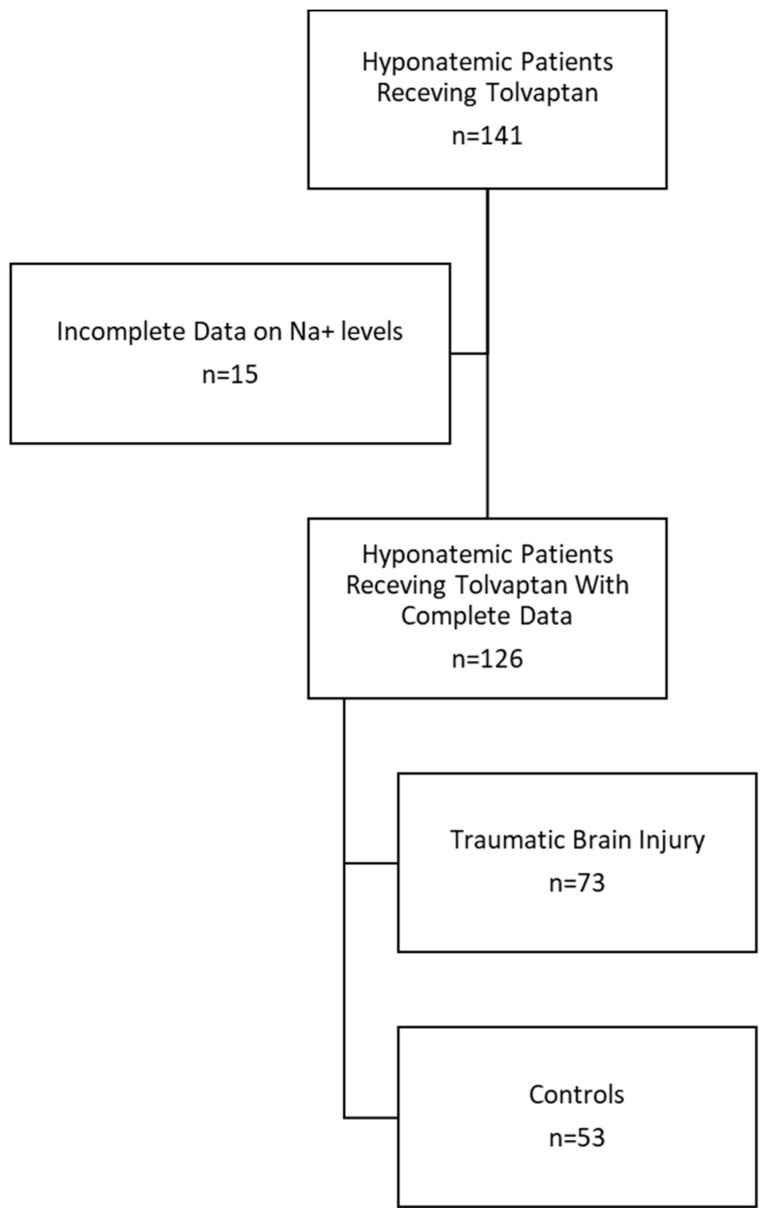
Patient selection.

Of the 141 adult patients in the ICU between January 2019 and November 2024 who were administered tolvaptan, 126 met the inclusion criteria and were included in the analysis (Figure 1). The non-trauma and trauma groups were similar in age and percentage of females. Among them, seventy-three patients were admitted with traumatic brain injury, and fifty-three patients were admitted for non-traumatic, chronic medical conditions and served as non-trauma controls (Figure 1, Table 1).

### 3.2. Outcomes

**Figure 2:**
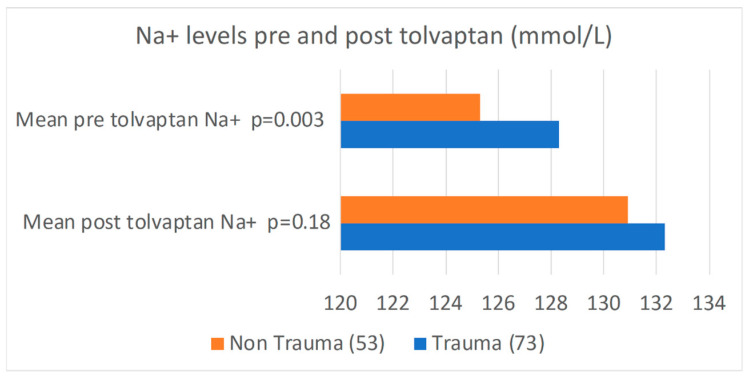
Horizontal bar graph demonstrating pre-tolvaptan and post-tolvaptan sodium levels after 48 h in both the non-trauma and trauma groups.

**Figure 3:**
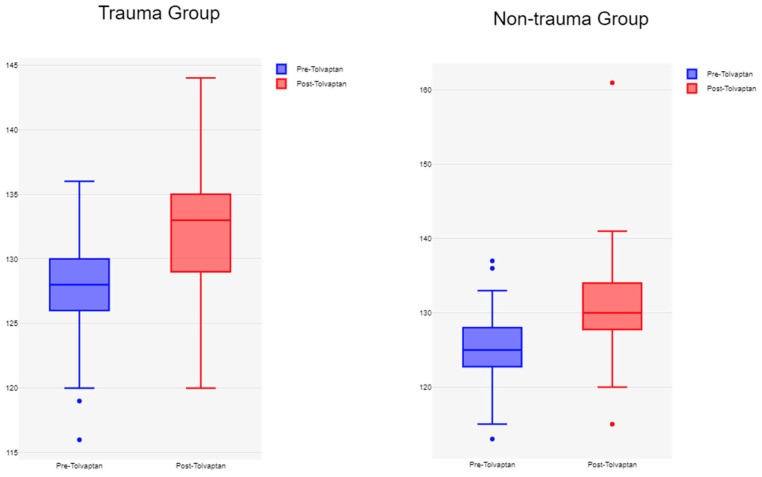
Box plot for serum sodium levels pre-tolvaptan and 48 h post-tolvaptan in both the trauma and non-trauma cohorts. (x axis—pre- and post-tolvaptan; y axis—serum Na+ level).

**Figure 4:**
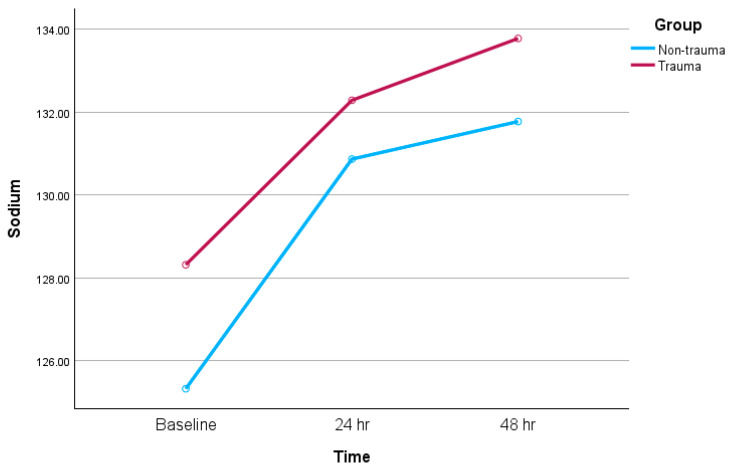
Line graph of serum sodium levels over 48 h. (x axis—time point of serum Na+ measurement; y axis—serum Na+ level).

Baseline sodium levels were higher in the trauma group compared to the non-trauma group (128.3 ± 4 mmol/L vs. 125.3 ± 5 mmol/L, *p* = 0.003) (Table 2, Figure 2). Following the administration of a median single dose of 15 mg of tolvaptan, both groups experienced a statistically significant increase in serum sodium levels at 24 and 48 h. Mean post-treatment sodium values at 48 h between the trauma and non-trauma cohorts were (133.8 ± 4 mmol/L vs. 131.8 ± 6 mmol/L (*p* = 0.036) (Table 2; Figure 3 and Figure 4). The majority of the patients experienced a positive sodium response to tolvaptan, with 95% of TBI patients and 87% of non-trauma patients demonstrating an increase in serum sodium within 48 h of the initial dose (*p* = 0.12) (Table 2). While the number of patients who achieved normalization of sodium (135–145 mmol/L) was significantly higher in the trauma group compared to the non-trauma group (48% vs. 30%, *p* = 0.045), after correcting for the differences in baseline sodium levels, there were no significant differences between the two groups [OR(95% CI) = 1.4 (0.61–3.14), *p* = 0.437].

### 3.3. Safety Data

Across both groups, tolvaptan was well-tolerated, with no reported cases of osmotic demyelination syndrome (ODS). The absence of ODS was confirmed by thorough clinical monitoring and, when indicated, radiological evaluation.

## 4. Discussion

In this retrospective cohort analysis, we evaluated the safety and efficacy of the use of tolvaptan in treating hyponatremia in patients with traumatic brain injury (TBI) compared to a non-trauma ICU population. Our study demonstrated that tolvaptan effectively increases serum sodium levels in both groups. After controlling for baseline sodium, there was a similar proportion of TBI patients achieving sodium normalization (135–145 mmol/L) within 48 h of administration. Notably, there were no documented cases of osmotic demyelination syndrome (ODS), suggesting a favorable safety profile for critically ill patients.

Hyponatremia is a common and serious complication in TBI patients, often due to SIADH. It is associated with increased intracranial pressure, cerebral edema, and poor neurological outcomes [1]. According to the Brain Trauma Foundation and the 2020 Neurocritical Care Society Guidelines for the Acute Treatment of Cerebral Edema, careful management of serum sodium is essential for avoiding additional brain injury [24,25]. Rajagopal et al. (2017) proposed a practical management protocol for TBI-associated hyponatremia, but most of these guidelines are limited on the direction provided for managing hyponatremia beyond hypertonic saline and mannitol when it is refractory [26]. The lack of definitive therapeutic guidelines for refractory hyponatremia in TBI patients emphasizes the need for further application of agents like tolvaptan. 

Our preliminary analysis suggests that tolvaptan effectively increases sodium levels in both TBI and non-trauma patients with hyponatremia. TBI patients demonstrated a similar rate of sodium normalization after tolvaptan treatment when compared to those without trauma. This study is one of the first studies to compare tolvaptan administration specifically in a TBI population when compared to a non-trauma cohort. This contributes important data to an understudied area of neurocritical care.

Tolvaptan, a selective vasopressin V2-receptor antagonist, blocks the binding of arginine vasopressin to V2 receptors, promoting the excretion of free water without losing serum electrolytes. This results in solute-free water excretion from the kidneys and ultimately helps correct the hyponatremia [7]. When considering alternatives to tolvaptan, fluid restriction remains first-line therapy for SIADH hyponatremia, but nearly half the patients do not respond adequately and require additional treatments [1]. Urea, which acts as an osmotic agent to induce free water clearance, is another treatment option that has been considered. However, a recent systematic review noted its variable efficacy, poor palatability, and limited patient adherence [27]. Furthermore, several recent studies have compared it to tolvaptan use. A randomized control trial by Delgado-Cuestra et al. (2025) found that both agents improved serum sodium levels, but tolvaptan led to a faster correction [28]. Similar studies showed that tolvaptan was more effective in achieving greater rates of sodium normalization than urea and easier to administer, although both had favorable safety profiles [29,30]. Other treatment options have limitations, such as hypertonic saline, which requires careful titration in the ICU with often the need for central line access. Additionally, many of these therapies can lead to overcorrection or volume overload if not carefully managed. However, with tolvaptan having a targeted mechanism of action, it lends itself to a favorable safety profile, which is particularly important in the TBI population when avoiding fluctuations in osmolality is crucial [31]. 

Our study was limited by its retrospective nature and single-center design, which may limit generalizability. The decision to administer tolvaptan was up to the treating physician and not part of a structured protocol. Additionally, we did not evaluate long-term outcomes such as mortality, length of stay, and long-term neurological recovery or effects. Furthermore, evaluating sodium levels beyond 48 h post-treatment would provide insight into the durability of the response and the recurrence rates of hyponatremia. 

Future studies are needed to confirm the safety and efficacy of tolvaptan use in TBI-associated hyponatremia and to identify optimal dosing strategies, timing, and duration of therapy. Additional evaluation should be performed to determine clinical outcomes such as neurological recovery, ICU and hospital length of stay, and mortality.

## 5. Conclusions

Our preliminary analysis suggests that tolvaptan effectively increases sodium levels in both TBI and non-trauma patients with hyponatremia. Further research is needed to fully characterize this response and determine the optimal use of tolvaptan for managing hyponatremia in the TBI population.

## Figures and Tables

**Table 1:** Baseline characteristics and outcomes.

Baseline Characteristics (Total Patients = 126)			
Patient Characteristic	Trauma (73)	Non-Trauma (53)	*p* Value
Age (years)	67.8 ± 18	71.1 ± 17	0.33
% females	42% (31)	49% (26)	0.46
Diagnoses	Injury Severity Scores	Admission Diagnoses Category	NA
	10 (4–16)	Renal	30.2% (16)
		Neurological	30.2% (16)
		Musculoskeletal	11.3% (6)
		Cardiac	13.2% (7)
		Pulmonary	3.8% (2)
		Others	11.3% (6)

**Table 2:** Effects of tolvaptan administration on both trauma and non-trauma patients.

Outcome of Tolvaptan Administration in Trauma and Non-Trauma Patients			
Measurement	Trauma (73)	Non-Trauma (53)	*p* Value
Mean pre tolvaptan Na+	128.3 ± 4	125.3 ± 5	0.003
Mean post tolvaptan Na+ (at 24 h)	132.3 ±5	130.9 ± 7	0.18
Mean post tolvaptan Na+ (at 48 h)	133.8 ± 4	131.8 ± 6	0.036
Median number of tolvaptan does	1 (1–3)	1 (1–3)	1
% patients with more than 1 Tolvaptan dose	10.9% (8)	56.6% (30)	<0.01
% of patients with increase in Na+ post-tolvaptan therapy within 48 h of first dose	95% (69)	87% (46)	0.12
% patients with decrease or no change in Na+ post tolvaptan therapy	5% (4)	13% (7)	0.12
Amongst patients with an increase in Na+, mean % increase in Na+ compared to before tolvaptan therapy	4.6% ± 2.7	6.5% ± 3.9	0.0018
% patients with normalization of Na+ (135–145) within 24 h of first dose	48% (35)	30% (16)	0.045
% patients with Na+ level ≥ 130	86% (63)	69.8% (37)	0.02
% patients with ≥ 8 meq increase in Na+ in 24 h	30% (22)	43.3% (23)	0.12

## Data Availability

Data may be made available upon reasonable request to the corresponding author.

## Abbreviations

The following abbreviations are used in this manuscript:

TBI	Traumatic Brain Injury
SIADH	Syndrome of Inappropriate Antidiuretic Hormone Secretion
AVP	Arginine Vasopressin
FDA	Food and Drug Administration
ICU	Intensive Care Unit
IRB	Institutional Review Board
CKD	Chronic Kidney Disease
CHF	Congestive Heart Failure
ODS	Osmotic Demyelination Syndrome
Na+	Sodium
SD	Standard Deviation
OR	Odds Ratio
CI	Confidence Interval
